# Preclinical studies and prospective clinical applications for bacteria-targeted imaging: the future is bright

**DOI:** 10.1007/s40336-016-0190-y

**Published:** 2016-07-16

**Authors:** Marjolein Heuker, Anna Gomes, Jan Maarten van Dijl, Gooitzen M. van Dam, Alexander W. Friedrich, Bhanu Sinha, Marleen van Oosten

**Affiliations:** 1 Department of Medical Microbiology, University of Groningen, University Medical Center Groningen, Hanzeplein 1, 9713 GZ Groningen, The Netherlands; 2 Department of Surgery, Division of Surgical Oncology, Nuclear Medicine and Molecular Imaging, Intensive Care, University of Groningen, University Medical Center Groningen, Hanzeplein 1, 9713 GZ Groningen, The Netherlands

**Keywords:** Imaging, Infection, Bacteria, Tracer, Fluorescence, Radioisotope

## Abstract

Bacterial infections are a frequently occurring and major complication in human healthcare, in particular due to the rapid increase of antimicrobial resistance and the emergence of pan-drug-resistant microbes. Current anatomical and functional imaging modalities are insufficiently capable of distinguishing sites of bacterial infection from sterile inflammation. Therefore, definitive diagnosis of an infection can often only be obtained by tissue biopsy and subsequent culture and, occasionally, a definite diagnosis even appears to be impossible. To accurately diagnose bacterial infections early, novel imaging modalities are urgently needed. In this regard, bacteria-targeted imaging is an attractive option due to its specificity. Here, different bacteria-targeted imaging approaches are reviewed, and their promising future perspectives are discussed.

## Introduction

Bacterial infections are of major concern both in hospital and community settings worldwide. Although much fundamental knowledge has been gained about micro-organisms and antimicrobial therapy, infections remain responsible for substantial patient morbidity and mortality these days [1]. In addition, infections become increasingly difficult to treat due to the rapid increase of antimicrobial resistance and the spread of pan-drug-resistant microbes [2, 3]. Besides the fact that drug-resistant infections are difficult to treat, their associated healthcare costs are substantially higher. For example, 1–2 % of the total joint arthroplasties will become infected, and the costs of treating such infections can amount up to $107.000 per case if caused by a resistant micro-organism. By comparison, the costs of treating infections with antibiotic-sensitive bacteria are substantially lower, revolving around $68.000 [4].

Despite a vast array of new technologies for the detection and typing of pathogens [5], diagnosing infections is often complex or even problematic. This results in a relatively, or even completely, blind management of infections. This complexity is a consequence of the fact that diagnosis is based on the combination of several nonspecific signs and symptoms, systemic inflammation markers, and visualization with fairly unspecific imaging techniques [6–8]. A definite diagnosis of infectious disease, with evidence of infection and identification of the causative microbial species, can only be obtained by culture and/or molecular detection. Ideally, this involves obtaining material directly from the infective focus, which often requires tissue biopsy for deep-seated infections. This invasive diagnostic procedure takes many hours or even several days to yield an answer and, occasionally, it is not even possible to obtain a representative biopsy. However, infections can be treated better when diagnosed accurately and early. Therefore, reliable and fast diagnostic processes are desirable [9].

Current imaging modalities to diagnose infectious disease comprise anatomical imaging modalities, such as computed tomography (CT), magnetic resonance imaging (MRI), and ultrasound (US), as well as functional imaging modalities, such as positron emission tomography (PET), single-photon emission computed tomography (SPECT), and scintigraphy. Unfortunately, these clinical imaging modalities by themselves are unable to differentiate bacterial infection from other infections (i.e., viral, fungal or parasitic), or from sterile inflammation [1, 10]. Ideally, an imaging modality would allow for a reliable detection of infection, differentiate infection from other causes of inflammation, and thereby circumvent the need of more invasive methods. Hence, a new imaging modality that allows for sensitive and specific imaging of bacterial infection, ideally even providing species and resistance information to guide optimal therapy, would be of high value in clinical practice. Such an imaging modality is most likely to be found in bacteria-targeted imaging approaches.

In this review, we present several bacteria-targeted imaging approaches and promising future perspectives of targeted imaging to diagnose infections.

## Methods

A literature search was performed in PubMed, searching for publications in English, in the period between January 1980 and February 2016. The following search terms and variations thereof were used: *imaging*, *detection*, *specific*, *targeted*, *bacterial*, *radionuclide*, *optical* and/or *fluorescence*. Of the publications thus retrieved, only those that were aimed at bacteria-specific in vivo imaging, in animals or humans, were selected. CT, MRI, and US were not included, because in a separate search using the terms *computed tomography*, *magnetic resonance imaging*, and *ultrasound*, *targeted*, *specific*, *bacterial*, *detection*, articles describing in vivo bacteria-specific imaging with CT, MRI or US were identified. Only those tracers closest to clinical introduction are reported.

### Targeted imaging

In recent years, an increasing interest in targeted imaging has been raised. Targeted imaging is based on an imaging agent, such as a radionuclide or fluorophore, coupled to a molecule (e.g., an antibody, an antibiotic, an antimicrobial peptide, a metabolizable compound, a bacteriophage, or a DNA/RNA-binding compound) that targets specific bacteria or other pathogens [9, 11]. Besides visualization of the site of infection, targeted imaging might also allow for the identification of the causative micro-organism. For additional information on the matter, we refer to the article of Mills et al. in this edition [11]. A more extensive review on targeted imaging was recently published by van Oosten et al. [9].

#### Radionuclide imaging

The functional non-targeted imaging modalities PET with ^18^F-fluoro-deoxy-glucose (FDG) and leukocyte scintigraphy are nowadays frequently used to track down sites of both infection and inflammation [12]. Imaging of infections with ^18^F-FDG–PET remains challenging due to the fact that all tissues with a high glucose uptake, such as brain, heart, malignancies, sterile inflammation and physiological wound healing, show increased FDG uptake [10]. Leukocyte scintigraphy partly overcomes this drawback as it allows to some extent the distinction between inflammation and infection by comparing images at different acquisition times [13]. However, a major drawback of leukocyte scintigraphy is that it is very laborious, since it involves the drawing of blood from the patient, harvesting and radiolabeling of leukocytes, and re-administering of the labeled leukocytes to the patient. Altogether, this procedure takes several hours. Other non-targeted radionuclide imaging techniques used to detect infections are ^67^Gallium-citrate imaging and bone scintigraphy [10]. In addition, two-step scintigraphy with streptavidin and ^111^Indium-biotin (^111^In-biotin) has been described by several research groups [14, 15]. The latter approach is based on the fact that streptavidin binds ^111^In-biotin with high affinity. However, this approach is unlikely to be bacteria-targeted as streptavidin accumulates at sites of infection as well as sites of inflammation [15].

The importance of non-invasive, specific, bacterial imaging in real time has become widely recognized. Most current knowledge on radionuclide-targeted imaging concerns ^99m^Technetium-ciprofloxacin (^99m^Tc-ciprofloxacin; Infecton^®^). In this regard, a large multi-centre clinical trial showed a sensitivity of 85.4 % and specificity of 81.7 % for detecting bacterial infections with ^99m^Tc-ciprofloxacin [16]. Although the sensitivity seems promising, its specificity is relatively low. Several other studies showed similar concern about the specificity of this imaging agent [17–19]. Indeed, ^18^F-labeled ciprofloxacin did not allow for bacteria-specific imaging in humans [20]. PET scans in four patients with bacterial soft tissue infections showed increased uptake of the tracer in infected areas. However, the signal was not retained in infected tissue and vanished at similar elimination half-life as in healthy tissue. It was, therefore, suggested that the radioactive signal was related to increased blood flow and vascular permeability in local infection [20]. Furthermore, there is a disadvantage of using ciprofloxacin due to the widespread resistance against this antibiotic [21, 22]. Besides ^99m^Tc-ciprofloxacin, other antibiotic-based tracers used in patients are radiolabeled ceftriaxone or fleroxacin [23, 24]. Indeed, ^99m^Technetium-labeled ceftriaxone allowed for successful visualization of infections in patients, but studies were too small to draw a final conclusion about the sensitivity and specificity of this tracer [23]. Also, many other antibiotics have been radiolabeled and tested, mainly in animal models [9].

As an alternative for labeled antibiotics, radiolabeled synthetic fragments of the antimicrobial peptide ubiquicidin can be used for detection of bacterial or fungal infections in patients. Ubiquicidin is a peptide originally isolated from mouse macrophages, and synthetic fragments of ubiquicidin were shown to bind to both Gram-positive and Gram-negative bacteria as well as to fungi. Small clinical trials investigating radiolabeled ubiquicidin showed a sensitivity of 100 % and a specificity from 80 to 100 % for detection of bacterial and fungal infections [25–28]. Altogether, ten successful clinical studies have been reported on specific imaging of infections with radiolabeled ubiquicidin [29]. Importantly, ^99m^Tc-ubiquicidin allowed the detection of infections with 93.7 % accuracy and with a pooled data sensitivity and specificity of 97.5 and 89 %, respectively. No immunological side effects were observed. Furthermore, a radionuclide-mediated tracer based on the nucleoside analog fialuridine [1-(2-deoxy-2-fluoro-β-d-arabino-furanosyl)-5-iodouracil; FIAU] has been evaluated in patients. It was shown that FIAU is taken up by bacteria and incorporated into their DNA, while this was not the case in human cells. Successful visualization of bacterial infections using ^124^I-labeled FIAU has been reported, with apparently no false-positive or false-negative results in seven patients [30]. In contrast, a recent study investigating the use of ^124^I-FIAU to image prosthetic joint infections in patients did not establish a clear correlation between the infection status and imaging results [31]. Moreover, two clinical studies addressing the use of ^124^I-FIAU were terminated because of poor image quality, and a lack of correlation between FIAU uptake and bone biopsy results (clinicaltrials.gov: NCT01705496 and NCT01764919). Therefore, the future role of radiolabeled FIAU in infection imaging is currently unclear.

Another extensively described approach, which is hardly used anymore, is the imaging of infections with radiolabeled human polyclonal immunoglobulin (HIG) [32–34]. HIG was supposed to accumulate at sites of infection, but it is apparently not bacteria specific since the reported specificity ranges from 50 to 90 %. This compromised specificity is mainly due to the fact that inflammation often results in a false-positive signal.

Not explored in humans so far, but promising as bacteria-specific imaging agents, are compounds that are exclusively metabolized by bacteria. Recently, detection of bacteria with the sugar 6-[^18^F]-fluoromaltose (^18^F-FM) has been reported [35]. Maltose and maltodextrins appear to be used as energy sources by the vast majority, if not all bacteria, since they express the maltodextrin transport complex in contrast to mammalian cells. ^18^F-FM, therefore, allows specific detection of all classes of bacteria and distinction of bacterial infection from other causes of inflammation. Another promising sugar for imaging purposes is sorbitol, which is a sugar alcohol mainly metabolized by Gram-negative bacteria, especially *Enterobacteriaceae*. 2-[^18^F]-fluoro-deoxy-sorbitol (^18^F-FDS) was shown to allow for the specific detection of infections with *Escherichia coli* or *Klebsiella pneumoniae* in mice [36]. Importantly, ^18^F-FDS neither accumulated in healthy nor malignant mammalian cells in vitro. Thus, ^18^F-FM, ^18^F-FDS and other labeled compounds that can only be metabolized by bacteria have a high potential for bacteria-targeted imaging and clinical translation.

#### Optical imaging

Use of optical (i.e., fluorescence) tracers for bacteria-targeted imaging is an upcoming and interesting topic nowadays. Optical imaging of infections seems highly feasible and has some important advantages over radionuclide imaging, such as (1) a high resolution, (2) the absence of ionizing radiation and its associated risks, (3) the possibility of real-time visualization, and (4) lower cost [37–39]. An important drawback of optical imaging is its limited penetration depth of maximally 1–2 cm using near infrared tracers, and even less for tracers with shorter wavelengths. This limited penetration depth makes fluorescence imaging suitable mainly for imaging of surfaces and superficially located structures, and thus solely applicable in superficially located infections, such as soft tissue or superficial implant infections, or in intra-operative applications [40].

Over the past decades, much experience has been gained in optical imaging, for example in the visualization of tumors in image-guided surgery [41–43]. Indeed, tumor-specific intra-operative fluorescence imaging of ovarian cancer was shown feasible in 2011 by targeting the overexpressed folate receptor-α in patients [44]. Especially intra- and peri-operative fluorescence imaging is likely to be of great value and major improvements, such as enhanced visual information during surgery and, as a consequence, improved sensitivity and specificity and more optimal resection margins, can be expected. In addition, deeper signal penetration might be feasible with improvements in fluorescence dyes and charge-coupled device (CCD) cameras.

**Fig. 1:**
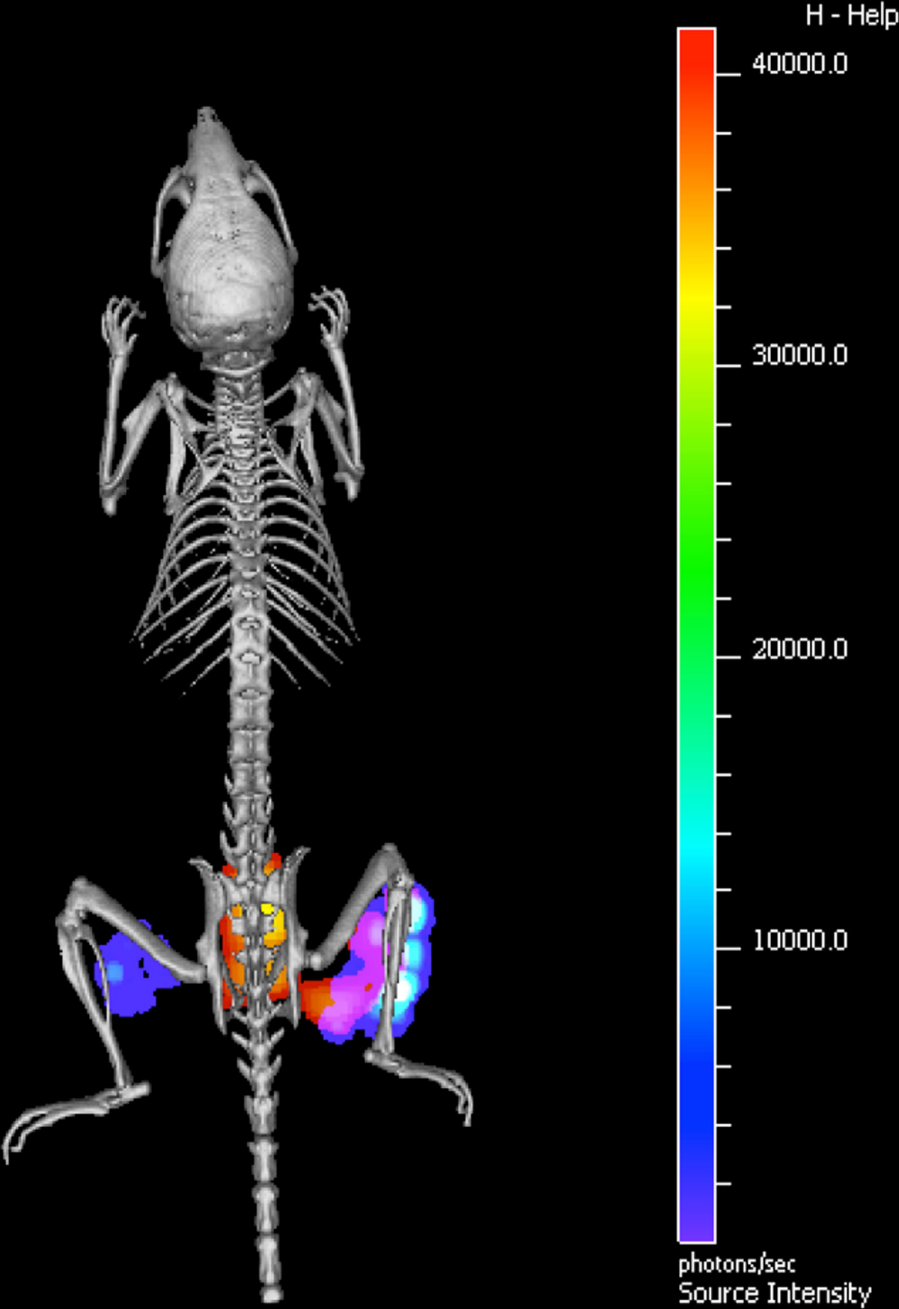
In vivo optical imaging of *Staphylococcus aureus* infection. Micro-computed tomography image of a mouse that was infected with a bioluminescent *S. aureus* strain in the right hind limb, and with a bioluminescent *E. coli* strain in the left hind limb. The NIR tracer vanco-800CW was administered intravenously [37]. The bioluminescence signal emitted by the infecting *S. aureus* and *E. coli* cells is depicted in *rainbow scale*, and the fluorescence signal due to vanco-800CW-binding in *red–yellow scale*. A clear co-registration of bioluminescence and NIR fluorescence was detected at the site of *S. aureus* infection. Moreover, a NIR fluorescence signal was detected in the bladder (in this image visible behind the spine). This bladder signal reflects the renal excretion of the tracer. Reprinted with permission from [37]

Despite the fact that many different fluorophores are available, only a few fluorophores are approved and tested for clinical use today, including fluorescein isothiocyanate (FITC; emission maximum 518 nm), indocyanine green (ICG; emission maximum 790 nm) and IRDye-800CW (emission maximum 800 nm). To date, bacteria-targeted fluorescence imaging is only described in a few in vivo studies, but with very promising results. First, Ning et al. showed specific uptake of fluorescently labeled maltodextrin by different bacterial strains and it was feasible to distinguish bacterial infections from sterile inflammation with high target-to-normal tissue (T/N) ratios in rats [45]. Second, Panizzi et al. used fluorescently and radiolabeled inactivated prothrombin, which binds to the staphylococcal coagulase produced by *Staphylococcus aureus*. These authors were able to make a distinction between *S. aureus*-induced versus *Staphylococcus epidermidis*-induced endocarditis, or sterile vegetations in mice [46]. Third, several research groups described the use of a fluorescent bis zinc (II)-dipicolylamine complex for infection imaging [47, 48]. This tracer attaches to the negatively charged membranes of bacteria. Unfortunately, the specificity of the fluorescent bis zinc (II)-dipicolylamine complex seems to be relatively low since this tracer also binds to negatively charged apoptotic cells [48]. Moreover, apoptotic cells are usually phagocytosed by macrophages which may further reduce the specificity. Lastly, non-invasive in vivo detection of infection caused by the Gram-positive bacterium *S. aureus* was shown using fluorescently labeled vancomycin [37]. Vancomycin-IRDye800CW (in short vanco-800CW) allowed the detection of *S. aureus* infection in vivo through several millimeters of tissue using a specialized CCD camera (Fig. 1). This was shown not only in a mouse model, but also in a human *post*-*mortem* model for biomaterial-associated infection. Vancomycin and IRDye-800CW are both approved substances for use in clinical practice, and it is therefore anticipated that vanco-800CW may be introduced safely for clinical use in the near future allowing for exploration of clinical applications. Whether the actual clinical application of vanco-800CW could be limited by increased vancomycin resistance in Gram-positive bacteria is currently unknown, but this is clearly a potential drawback of all antibiotic-based tracers [9].

#### Optoacoustic imaging

The relatively new detection technology of optoacoustic imaging has been recently suggested for use in clinical imaging. Like optical imaging, this technique is based on absorption of light of a certain wavelength by an appropriate fluorophore coupled to a specific targeting molecule. After absorption of light, the fluorophore molecules will undergo thermo-elastic expansion, which leads to the emission of ultrasonic pressure waves. In optoacoustic imaging, these ultrasonic waves are detected by specialized sensors [49]. Optoacoustic imaging allows for deeper visualization in tissue (around 11 cm in muscle tissue), since ultrasonic waves have longer wavelengths and deeper penetration than light [50]. This deeper penetration can particularly be valuable in the visualization of, e.g., bacterial infection of biomaterials or endocarditis. Next to these advantages, optoacoustic imaging offers a high resolution and high contrast comparable to that of MRI [49].

#### Other imaging modalities

Specific imaging with CT and MRI scanning has been reported for imaging of malignancies, using targeted modality-specific contrast agents [51, 52]. To the best of our knowledge, examples of bacteria-targeted imaging with CT or MRI in vivo have not yet been documented [9]. Neither were reports found addressing bacteria-specific targeting in vivo with the gas-filled microbubbles that are used as contrast agents in US [9]. Nevertheless, Anastasiadis et al. showed feasibility of a combined optical and acoustic evaluation in vitro, using targeted encapsulated gas bubbles for detection of early- and late-stage biofilm formation, potentially allowing for biofilm-specific imaging with US in vivo [53].

### Smart activatable tracers

**Fig. 2:**
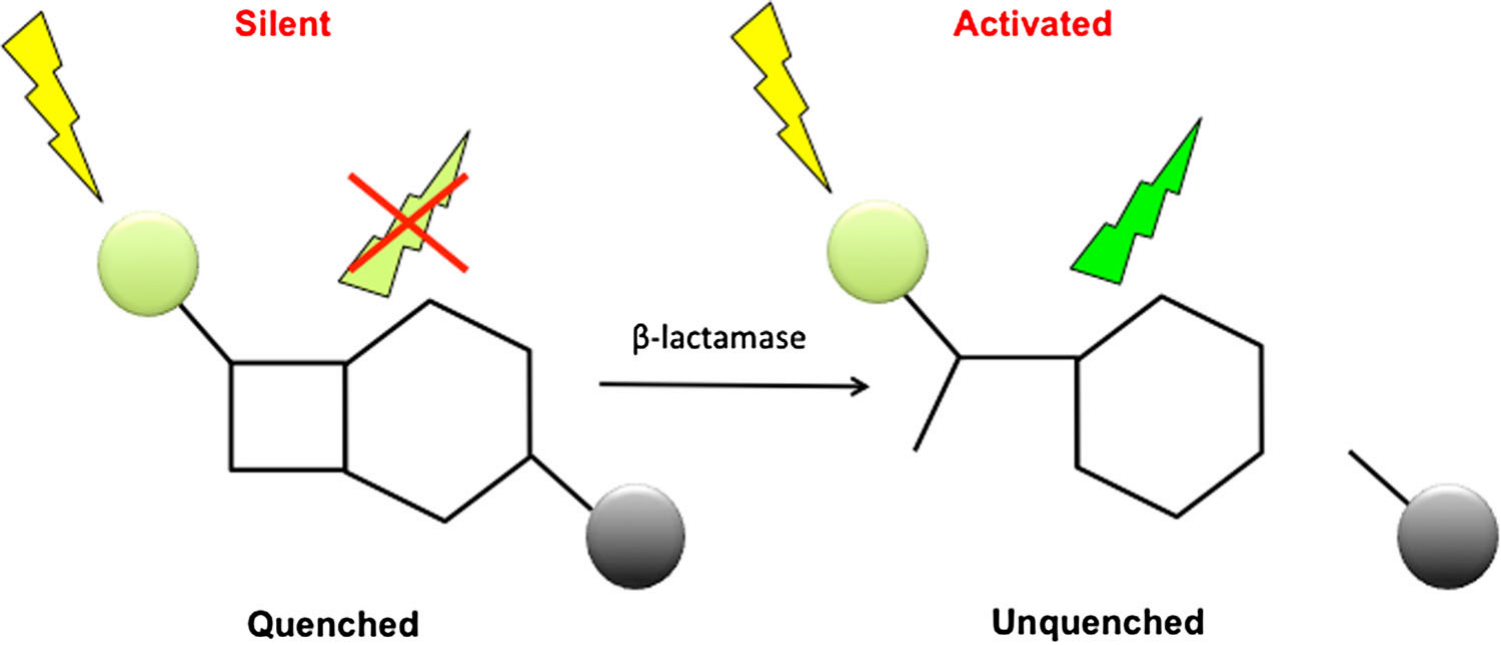
Mechanistic principle of β-lactam-based smart-activatable tracers. The intact tracer does not emit light due to the presence of a quencher (*gray sphere*). After hydrolysis of the β-lactam ring by a β-lactamase, the quencher is detached from the fluorophore (*green sphere*) and the tracer emits light

An exciting approach in the field of infection-targeted imaging is the detection of bacteria with the so-called “smart activatable tracers” (Fig. 2). Smart activatable tracers are optical tracers that are quenched in their normal state, and thus do not emit any signal. When the tracer encounters its target, the tracer is cleaved by bacterial enzymes, resulting in removal of the quencher. Subsequently, a fluorescence signal is emitted that can be detected. Imaging with smart activatable tracers usually results in a higher T/N ratio as compared to conventional tracers, and consequently in more efficient imaging. Successful use of smart activatable tracers has been reported for imaging tumors, as well as bacteria [54–56]. However, for infection imaging, results have so far only been obtained in animal studies. Kong et al. designed a smart activatable tracer based on a β-lactam ring, which is hydrolyzed by bacterial β-lactamases, leading to activation of the fluorophore [55]. Successful imaging with this tracer has been shown for *Mycobacterium tuberculosis* infection in mice. Although only *M. tuberculosis* infection imaging has been described, this tracer might be applicable for all β-lactamase-producing bacteria. No T/N ratios have been reported yet for this β-lactam smart activatable tracer and, thus it is not clear whether the smart activatable tracer in this case indeed offers the increased T/N ratios required.

Furthermore, Hernandez et al. reported the non-invasive detection of *S. aureus* infection in mice, with an intravenously administered nuclease-activated tracer [56]. The quenched fluorescence oligonucleotide tracer is specifically activated by micrococcal nuclease (MN), a nuclease secreted by *S. aureus*. This tracer was proven to be MN- and thus *S. aureus*-specific, the mutated nuclease-deficient *S. aureus* showed a significantly lower signal. For this tracer, good T/N ratios have been reported.

### Multi-modality tracers

A combination of imaging modalities allows for the concurrent application of the advantageous properties of each single modality, and thereby optimization of diagnosis. Multi-modality tracers are based on a targeting molecule, coupled to two or more imaging modality agents, such as radionuclides, fluorophores, CT contrast agents, MRI contrast agents, or microbubbles [57]. Such a multi-modality tracer allows for imaging of a target with one single tracer by two or more different imaging modalities simultaneously. For example, this potentially offers an opportunity for pre-operative detection of infection and tracking down its localization and, subsequently, it offers a possibility for optical visualization during invasive procedures. Such multi-modality tracers have been described for targeting inflammation (e.g., CD20 on B-cells, integrins and matrix metalloproteinases) [58]. Notably, a bacteria-targeted multi-modality tracer based on the antimicrobial peptide ubiquicidin has recently been described [59]. Ubiquicidin was conjugated to a radioisotope and fluorophore and this dual-modality tracer was able to detect *S. aureus* and *K. pneumoniae* infections in vivo using a combined imaging system. In our opinion, these multi-modality tracers are potentially of great value in bacterial infection imaging.

### Photodynamic therapy

Besides using targeted modalities to optimize diagnosis of infection, targeted therapy could be used to improve infection treatment. In particular, targeted photodynamic therapy (PDT) could play an important role in the treatment of infections. PDT is based on excitation of photosensitive molecules with light of a certain wavelength, resulting in an optical signal as well as cytotoxic molecules that are capable of destructing the targeted cells [60, 61]. Treatment characteristics of this approach are promising, but untargeted PDT leads to damage of healthy tissue due to the non-specific uptake of photodynamic molecules, currently limiting this form of PDT to skin and dental infections [60, 62]. Targeted PDT might solve this drawback and was already shown to be successful in cancer treatment [61, 63]. It seems conceivable that targeted PDT could play an important role in the treatment of bacterial infections as well. Unfortunately, PDT would be limited to localized, easily accessible or superficial infections due to a limited penetration depth of available lasers. To date, there is little experience with targeted PDT in infections in vivo. Ragàs et al. showed eradication of methicillin-resistant *S. aureus* (MRSA) infection in a murine burn wound model [64]. A cationic photosensitizer, which was topically administered, was used to target the bacteria. Although this technique eradicated MRSA, it is questionable whether this tracer is really “targeted”, since the photosensitizer binds to the negatively charged bacterial cell walls based on the cationic molecules, which probably would also have affinity for other negatively charged structures, such as apoptotic tissue. There is some more experience with targeted PDT in in vitro infection models. In this regard, feasibility has been shown for antibody-directed and antimicrobial peptides-directed PDT [65–67]. Suci et al. described successful targeted PDT based on a biotinylated photosensitizer and a biotinylated antibody specific for *Aggregatibacter actinomycetemcomitans*, coupled by streptavidin [68]. These results seem promising for the treatment of periodontal biofilms. Overall, PDT seems to be an interesting diagnostic and therapeutic application for locoregional infections.

### Potential clinical applications of targeted imaging

Targeted imaging could enable earlier detection of infections, resulting in earlier institution of an accurate treatment. The more accurate treatment will subsequently represent a crucial element in the fight against bacterial resistance. Besides the application of targeted imaging in pre-operative diagnostics and post-operative follow-up of infections, it could also be used for intra-operative surgical guidance, theoretically leading to a more optimal resection of infected tissue and minimized damage in healthy tissue.

Potential use of tracers for different infections is outlined in Table 1.

**Table 1:** Overview of infections, with most common causative micro-organisms, and potentially suitable tracers for detection

Infection	Common causative micro-organisms (>10 %)	Potential tracers for detection	Remarks
Necrotizing fasciitis	*Streptococcus pyogenes*, other β-hemolytic streptococci, *Staphylococcus aureus*; (an)aerobic mixed flora, *Clostridium perfringens* ^a^, other clostridia^a^	CEF, CIP, FIAU, MALT, UBI, VAN	
Septic arthritis	*Staphylococcus aureus*, streptococci, *N. gonorrhoeae*	CEF, CIP, FIAU, FLER, MALT, NUC, PRO, UBI, VAN	
Infective endocarditis	*S. aureus*, coagulase negative staphylococci, viridans streptococci, enterococci, HACEK organisms^a^	CEF, CIP, FIAU, MALT, NUC, PRO, UBI, VAN	
Infected surgical meshes	*S. aureus*, coagulase negative staphylococci, streptococci, enterococci	CEF, CIP, FIAU, MALT, NUC, PRO, UBI, VAN	
Infected surgical grafts and meshes	*S. aureus*, coagulase negative staphylococci, streptococci, enterococci	CEF, CIP, FIAU, MALT, NUC, PRO, UBI, VAN	
Meningitis	*Streptococcus pneumoniae*, *Neisseria meningitidis*, *Haemophilus influenzae*, *Listeria monocytogenes* ^a^	CEF, FIAU, MALT, UBI	The tracer has to cross the blood–brain barrier
Bacteremia	Miscellaneous	CEF, CIP, FIAU, MALT, NUC, PRO, SOR, UBI, VAN, BLA	
Pneumonia	*Streptococcus pneumoniae*, *Haemophilus influenzae*, *S. aureus*. Hospital-acquired pneumonia: miscellaneous	CEF, CIP, FIAU, MALT, NUC, PRO, SOR, UBI, BLA	

Tracers can be suitable in two possible ways, namely (1) detection of infection by targeting the vast majority of causative pathogens, or (2) by detection of particular species, which would have direct implications for the choice of therapy. Ideally, future approaches would offer both possibilities. Potentially usable tracers not only include the tracers in their current published structures but also future variants (e.g., modification from radiolabeled to optically labeled, or vice versa). Microorganisms and tracers are mentioned in alphabetical order

^a^Micro-organisms that occur in less than 10 % of the cases, but are classically associated with the respective infection. The specific tracers listed are: ^99m^Tc-ceftriaxone (CEF), ^99m^Tc-ciprofloxacin/^18^F-ciprofloxacin (CIP), ^124^Iodine-FIAU (FIAU), ^18^F-fleroxacin (FLER), 6-[^18^F]-fluoromaltose/maltodextrin-based optical tracer (MALT), activatable nuclease-targeted optical tracer (*S. aureus*-directed; NUC), prothrombin-based optical/radiolabeled tracer (PRO), 2-[^18^F]-fluoro-deoxy-sorbitol (SOR), ^99m^Tc-ubiquicidin (UBI), vancomycin-IRDye 800CW (VAN), activatable β-lactamase-targeted optical tracer (BLA). HACEK organisms (fastidious Gram-negative bacilli): *Haemophilus* spp., *Aggregatibacter actinomycetemcomitans*, *Cardiobacterium hominis*, *Eikenella corrodens*, *Kingella kingae/Kingella denitrificans*

#### Necrotizing fasciitis

Necrotizing fasciitis is a rapidly progressive infection of the deeper layers of skin and subcutaneous tissues, which requires immediate aggressive surgery and antibiotic therapy. To date, surgeons mainly rely on visual and tactile information during surgery. Infection borders of necrotizing fasciitis are difficult to recognize and can only be analyzed during surgery using frozen sections. Unfortunately, this is an indirect method giving information only about the small site of tissue where a biopsy was taken. Clearly, it is desirable to develop new methods that provide the needed information in real time and intra-operatively. Using targeted imaging, surgeons can be provided with extra visual information. In targeted optical imaging of ovarian cancer in patients, surgeons were able to detect small tumor spots of <1 mm [69]. Hypothetically, intra-operative targeted imaging can also result in better detection of infection, and more precise and radical resection, while sparing as much as possible healthy tissue. This may eventually lead to a better prognosis for patients with less mutilating outcomes. Presumably, targeted imaging would be of great value for necrotizing fasciitis-related surgery in the future.

#### Septic arthritis

Septic arthritis is most commonly caused by bacteria, but also fungi or viruses can be the causative agents. Notably, also sterile inflammatory diseases, such as rheumatoid arthritis or gout, are very common causes of arthritis. Arthritis caused by bacterial pathogens is often rapidly destructive, and thereby of the most fulminant subtype. In daily practice, the causative agent of arthritis is difficult to establish from the outside, and arthrocentesis is used to collect synovial fluid. Subsequent microbiological culturing is needed to find evidence of infection and to identify the responsible bacteria. Minimal-invasive targeted imaging techniques would be of great importance in this clinical situation, to (1) quickly and adequately distinguish bacterial arthritis from other pathologies, and (2) start early with appropriate antibiotic therapy. This may lead to a better prognosis for patients and a lowered chance of eliciting bacterial resistance.

#### Infective endocarditis

Infective endocarditis (IE) causes serious morbidity and mortality (40 % of all patients die in 1 year) [70]. Therefore, early and accurate diagnosis is crucial. Unfortunately, diagnosing IE remains a challenge in current clinical practice, due to its variable clinical presentation of both cardiac and extra-cardiac manifestations. Diagnosis of IE is largely based on the modified Duke criteria, of which blood cultures and echocardiography are cornerstones [71]. These criteria have only a sensitivity and specificity of around 80 % when no artificial material is involved [71], and the ultimate diagnosis has still to be made by expert clinical judgement. Echocardiography has been shown to miss life-threatening complications in 30 % of patients [72]. In addition, blood cultures show no causative micro-organism in up to 31 % of all cases of IE [73], partly due to prior antimicrobial therapy. This poses considerable diagnostic and therapeutic dilemmas in clinical practice, as anatomic information guides indication and timing of surgical treatment. Furthermore, it is essential to determine the causative micro-organism and its resistance pattern to implement appropriate antimicrobial therapy. Here, opportunities for improved imaging are, obviously, highly desirable. Promising results in this direction have been published using conventional ^18^F-FDG–PET/CT and leukocyte scintigraphy [74]. These functional techniques use, respectively, ^18^F-FDG as tracer and PET/CT for detection, or radiolabeled white blood cells (e.g., with ^99m^Tc-HMPAO) as tracers and SPECT/CT for detection. Pizzi et al. have reported the largest study to date evaluating ^18^F-FDG–PET/CT in endocarditis. Their results show that the largest group of 50 patients with possible endocarditis (54 %) could be reduced to 5 (5 %) [75]. Furthermore, Rouzet et al. compared both imaging techniques in IE, and their results mainly indicate that ^18^F-FDG–PET/CT is more sensitive, whereas leukocyte scintigraphy is more specific [76]. However, even when taking most recent (non-specific) imaging developments into account [74], more specific diagnosis of IE could prove critical in a vast amount of remaining cases with diagnostic uncertainty. In addition, opportunities to accelerate accurate diagnosis and treatment could prove lifesaving. Therefore, targeted imaging represents an appealing option for IE diagnosis. For example, targeted imaging could disclose the pathogenic bacterium in cases of negative blood cultures, so that adequate antibiotic therapy can be given. Besides, targeted imaging could point towards additional and distant sites of cardiac infection, which should be taken into account in the individualized therapy plan. On top of this, intra-operative targeted imaging could visualize the exact borders of infected tissue of which radical resection could potentially improve patient outcome. This is likely to be highly important in this condition since it is mandatory to immediately implant prosthetic material in the area of infected tissue.

#### Infected surgical meshes

Surgical meshes are commonly used to support tissue. In particular, these meshes are useful for permanent support in the repair of abdominal and inguinal hernias. Occasionally, a wound infection will occur after surgical intervention and, on longer term, intestinal-cutaneous fistulas can occur after abdominal surgery. Often it is difficult to determine whether and to what extent implanted meshes are involved in these infectious processes. This is highly relevant to predict prognosis and determine most optimal therapy. Targeted imaging could provide information on (1) whether the mesh is involved in the infectious process, and (2) what is the causing bacterial species. Due to the minimal-invasive targeted imaging technique, a more invasive technique, with ultimately preventive removal of the mesh, may be avoided.

#### Infected vascular grafts

Superficial (i.e., dialysis shunts) and deeper seated (e.g., aortic aneurysm repair) vascular graft infections are responsible for high patient morbidity and mortality, if not treated immediately [77]. Frequently, only the outside of the graft is infected resulting in negative blood cultures. Nowadays, ^18^F-FDG–PET-scan and/or leukocyte scintigraphy are used for the diagnosis of vascular graft infections in addition to the CT-scan. Both give information about inflammatory activity, in addition to the anatomic information derived from a simultaneous and/or separate CT-scan. However, infection cannot be reliably distinguished from sterile inflammation or physiological wound healing using ^18^F-FDG–PET nor CT-scan. Therefore, early infected vascular grafts cannot always be readily detected [78]. Also, notwithstanding its high sensitivity (91 %), ^18^F-FDG–PET is not an ideal imaging approach for detection of infected vascular grafts since its specificity is rather low (64 %) [77]. Leukocyte scintigraphy seems to allow for a higher specificity, especially early post-operatively, and is recommended if the CT-scan is inconclusive [79, 80]. Nevertheless, a non-invasive specific targeted imaging technique is highly desirable for detection of infected vascular grafts. The most important advantage of such a targeted approach is that an invasive surgical procedure is not needed to diagnose bacterial infection, to determine which vascular grafts are involved in infection, and to what extent these grafts are affected. Targeted imaging that allows for determination of the causative bacterial pathogen would highly aid in directing optimal therapy. This would be especially helpful in detecting fastidious micro-organisms that cannot be routinely cultured, such as *Coxiella burnetii*. Besides, intra-operative targeted imaging could aid for determining the extent of infected tissue and grafts, and for determining the most optimal resection borders.

#### Meningitis

The diagnosis of meningitis is currently based on blood tests (i.e., C-reactive protein and complete blood count), blood cultures, and most importantly examination of the cerebrospinal fluid (CSF). CSF should be obtained of every patient with a suspicion of meningitis to identify the causative micro-organism unless a lumbar puncture is contraindicated (e.g., brain tumor, abscess, or a high intracranial pressure). In selected patients a CT scan or MRI scan is recommended prior to lumbar puncture to check for the existence of contraindications, which delays the actual puncture. As it is of great importance to treat meningitis quickly with antibiotics, in these cases broad-spectrum antimicrobial treatment is commonly initiated before lumbar puncture to prevent treatment delay. Unfortunately, this early start of antibiotic treatment often interferes with finding the causative agent, whereas culture of CSF that is obtained after start of antibiotic treatment has a substantially lower sensitivity. A sensitive imaging modality to detect meningitis at an early stage and to discriminate between different causative pathogens would be highly desirable [81]. Especially the new hybrid PET/MRI approach seems very promising for distinguishing meningitis from other pathologies such as abscesses and encephalitis [82–84]. This might even allow a distinction of meningitis caused by viruses from bacterial meningitis by pattern recognition. Bacteria-targeted PET tracers, such as ^18^F-FDS or ^18^F-FM, which detect a subset or all bacterial species, could more clearly distinguish bacterial infections from other pathologies, and especially tracers that identify particular bacterial groups or even species could enable quick and more accurate treatment. An extra challenge might be found in designing tracers that reliably pass the blood–brain barrier upon intravenous administration. Also, it remains to be seen whether the bacterial load in meningitis is high enough for detection with the current PET cameras, which have a relative low resolution (3–4 mm). Clearly, a rapid and highly sensitive test for the presence of bacteria using fluorescence tracers could improve diagnostic accuracy and accelerate diagnosis in patients where CSF has been obtained. This option could make use of optical smart activatable tracers and dedicated sensors which might be even used as bedside tests.

#### Bacteremia

The gold standard for diagnosis of bacteremia (in case of sepsis or endocarditis) is the isolation of micro-organisms from a blood culture. Ideally, blood cultures are obtained prior to antimicrobial therapy. Unfortunately, it takes several days of culture until the causative micro-organism with its matching resistance pattern is identified. In case of infection with a fastidious micro-organism or antibiotic usage prior to the blood culture collection, identification of the bacterial species can be problematic. Real-time detection of bacteria in the blood stream of a patient would, therefore, be of great value. In particular, optical targeted imaging might be ideal for this application, since this makes real-time bedside measurements feasible. For instance, the diagnosis of infection could potentially be made much faster by implementing continuous measurement systems. This could involve optical smart activatable tracers and dedicated sensors. Subsequently, this would also allow for treatment follow-up, to detect whether the bacterial load in the blood stream is decreasing.

#### Pneumonia

Currently, diagnosis of pneumonia comprises clinical symptoms and signs, radiography and sputum culture. Radiography reveals in most cases pulmonary infiltrates, but differentiation between infection and other causes of pulmonary infiltration (e.g., atelectasis, infarction, hemorrhage, acute respiratory distress syndrome and malignancy) remains difficult with a specificity of only 33–70 % [85, 86]. Sputum culture, or even more optimal broncho-alveolar sample culture, can provide relevant information, but it takes several days before a reliable identification of the causative agent and its drug-resistance profile is available, if at all. Besides, representative respiratory material is often not obtainable due to the clinical situation of the patient. As a consequence, physicians substantially overtreat patients by early empirical initiation of broad-spectrum antibiotic therapy. Thus, there is currently an unmet need for fast and accurate diagnosis of pneumonia and identification of its causative agent to allow narrow-spectrum treatment and avoid unnecessary antibiotic usage. Development of a set of infection-targeted optical tracers for direct detection of pneumonia is a highly promising approach. First clinical trials on bacteria-targeted imaging are currently initiated at the Royal Infirmary in Edinburgh to detect bacterial pneumonia using a bronchoscope combined with confocal laser endomicroscopy (clinicaltrials.gov: NCT02558062 and NCT02491164). Potentially, this approach can visualize pneumonia in situ, not only providing direct cues about the presence and localization of pneumonia, but also offering the possibility of focused sample collection for subsequent microbiological analysis (i.e., microscopy, culture or molecular analysis).

## Conclusion

Bacteria-targeted imaging is of significant upcoming interest. Progress has been made in the development of specific tracers, as well as in the development of imaging modalities to visualize infection. It remains difficult to predict which tracers or modalities will prove most appropriate for clinical use, but it is to be expected that some of the approaches described in this review will eventually find their place in routine clinical diagnostic settings. The further implementation of new imaging techniques, such as multi-modality imaging or optoacoustic imaging, and smart activatable tracers, holds even greater promise for quick and accurate detection of infections. This may ultimately be extended to antibacterial therapy in case of targeted PDT. We thus conclude that bacteria-targeted imaging has a bright future.
